# Nitrogenase Gene Amplicons from Global Marine Surface Waters Are Dominated by Genes of Non-Cyanobacteria

**DOI:** 10.1371/journal.pone.0019223

**Published:** 2011-04-29

**Authors:** Hanna Farnelid, Anders F. Andersson, Stefan Bertilsson, Waleed Abu Al-Soud, Lars H. Hansen, Søren Sørensen, Grieg F. Steward, Åke Hagström, Lasse Riemann

**Affiliations:** 1 Department of Natural Sciences, Linnaeus University, Kalmar, Sweden; 2 Science for Life Laboratory, School of Biotechnology, KTH Royal Institute of Technology, Stockholm, Sweden; 3 Department of Ecology and Genetics, Limnology, Uppsala University, Uppsala, Sweden; 4 Section for Microbiology, Department of Biology, University of Copenhagen, Copenhagen, Denmark; 5 Department of Oceanography, School of Ocean and Earth Science and Technology, University of Hawaii, Honolulu, United States of America; 6 Marine Biological Section, Department of Biology, University of Copenhagen, Helsingør, Denmark; Argonne National Laboratory, United States of America

## Abstract

Cyanobacteria are thought to be the main N_2_-fixing organisms (diazotrophs) in marine pelagic waters, but recent molecular analyses indicate that non-cyanobacterial diazotrophs are also present and active. Existing data are, however, restricted geographically and by limited sequencing depths. Our analysis of 79,090 nitrogenase (*nifH*) PCR amplicons encoding 7,468 unique proteins from surface samples (ten DNA samples and two RNA samples) collected at ten marine locations world-wide provides the first in-depth survey of a functional bacterial gene and yield insights into the composition and diversity of the *nifH* gene pool in marine waters. Great divergence in *nifH* composition was observed between sites. Cyanobacteria-like genes were most frequent among amplicons from the warmest waters, but overall the data set was dominated by *nifH* sequences most closely related to non-cyanobacteria. Clusters related to Alpha-, Beta-, Gamma-, and Delta-Proteobacteria were most common and showed distinct geographic distributions. Sequences related to anaerobic bacteria (*nifH* Cluster III) were generally rare, but preponderant in cold waters, especially in the Arctic. Although the two transcript samples were dominated by unicellular cyanobacteria, 42% of the identified non-cyanobacterial *nifH* clusters from the corresponding DNA samples were also detected in cDNA. The study indicates that non-cyanobacteria account for a substantial part of the *nifH* gene pool in marine surface waters and that these genes are at least occasionally expressed. The contribution of non-cyanobacterial diazotrophs to the global N_2_ fixation budget cannot be inferred from sequence data alone, but the prevalence of non-cyanobacterial *nifH* genes and transcripts suggest that these bacteria are ecologically significant.

## Introduction

The availability of nitrogen (N) limits biological production in vast areas of the global ocean and is therefore tightly linked to the fixation of atmospheric carbon dioxide and export of carbon from the ocean's surface [1]. A proper understanding of the marine N cycle is therefore needed for accurate quantification and forecasting of oceanic carbon cycling. The major processes controlling the inventory of bioavailable N in the ocean are fixation of N_2_ gas, which supplies new N to the food web, and denitrification and anaerobic ammonia oxidation, which remove it. Using best current estimates for each process, the calculated global oceanic N budget is out of balance with losses exceeding inputs [2], possibly as a consequence of incomplete knowledge about the identity, diversity, and autecology of N_2_-fixing microorganisms (diazotrophs; [3]). Hence, it is pertinent to address the composition and ecological dynamics of diazotrophs in the global ocean in order to understand, and ultimately predict, ecosystem productivity and carbon dynamics.

N_2_ fixation is exclusively performed by members of the Domains *Bacteria* and *Archaea* [4]. The filamentous cyanobacterium *Trichodesmium* [5], and cyanobacterial symbionts of diatoms (*Richelia*; [6]) were long thought to be the only important diazotrophs in the ocean. However, molecular analyses targeting the *nifH* gene, encoding the conserved iron–protein subunit of the nitrogenase enzyme complex, led to the realization that unicellular cyanobacteria are also significant contributors to N_2_ fixation in tropical and subtropical oceans [7]. Phylogenetic analyses suggest that diverse non-cyanobacteria are also widespread and actively expressing the *nifH* gene in marine waters, though, this information is primarily based on small *nifH* cDNA clone libraries with restricted geographic coverage [7]–[9].

In the present study, we sought to obtain a broad overview of marine planktonic diazotrophs by deep sequencing of nitrogenase gene libraries prepared from surface ocean water collected at ten locations world wide representing nine distinct biogeographical provinces. Available data indicate that only a fraction of the putative diazotrophs in marine plankton express nitrogenase at any given time [10]. Thus, to have the most complete inventory of microorganisms with the potential to fix N_2_, we chose to focus on DNA samples. RNA samples were also analysed at two of the ten locations in order to evaluate which genes were also expressed at a given time. In this first in-depth sequencing of *nifH* PCR amplicons from marine waters, we document a large diversity and spatial variation of putative diazotrophs and a striking dominance by members of the Phylum *Proteobacteria*. Our findings call for further interrogation of the ecological role of non-cyanobacteria in marine N cycling.

## Materials and Methods

### Sample collection, nucleic acids extraction and cDNA synthesis

**Figure 1:**
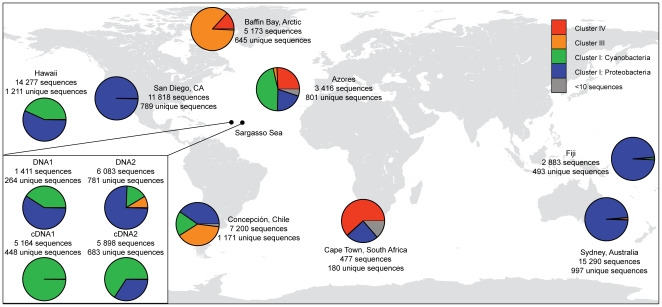
World map of sampling locations showing the distribution of *nifH* Clusters. Pie charts display the distribution of *nifH* Clusters within each sample. Clusters containing <10 sequences (shown in grey) were not phylogenetically designated. Note that Cluster I is split into *Proteobacteria* and Cyanobacteria, but that Cluster III also contains some *Proteobacteria*. For the Sargasso Sea samples, which were prefiltered (10 µm) to avoid filamentous cyanobacteria, the pie charts for DNA and cDNA samples are shown in the bottom left corner.

Surface open seawater samples (5 m depth) from ten locations (Figure 1, Table 1) were collected on three occasions; in the Arctic (Baffin Bay, July 2007), the Sargasso Sea (two locations, ∼5 km apart, May 2008) and during two concurrent sampling campaigns (from May 20^th^ to June 3^rd^, 2003) in the Atlantic Ocean offshore of the Azores (Portugal) and of Cape Town (South Africa); in the Pacific Ocean offshore of San Diego (California), Honolulu (Hawaii), Sydney (Australia), Concepción (Chile), and the Fiji Islands [11]. Data on temperature, chlorophyll *a* (chl *a*) and bacterial abundance were obtained [11]. At each station, 1–6 L of seawater was filtered onto a 0.2 µm (47 mm) Supor filter (PALL Corporation) or a 0.22 µm Sterivex filter (Millipore) and immediately frozen in 1 mL TE buffer (10 mM Tris-HCl, 1 mM EDTA, pH 8.0). For RNA samples, 500 µl RNAlater solution (Ambion) was added instead of TE buffer. To avoid dominance by *Trichodesmium* in the Sargasso Sea samples, a 10 µm polycarbonate prefilter was used (GE Water & Process Technologies).

**Table 1:** Environmental data and sample locations.

Sampling location	Baffin Bay, Arctic	Azores	Cape Town, South Africa	Sydney, Australia	Fiji	Honolulu, Hawaii	San Diego, CA	Concepción, Chile	Sargasso Sea 1	Sargasso Sea 2
Sampling date (dd/mm/yyyy)	14/07/2007	20/05/2003	20/05/2003	25/05/2003	28/05/2003	02/06/2003	28/05/2003	03/06/2003	13/05/2008	15/05/2008
Sampling coordinates	71°33′N 65°23′W	38°25′N 28°48′W	34°15′S 17°53′E	34°07′S 151°12′E	18°10′S 178°12′E	21°10′N 157°55′W	32°53′N 117°23′W	36°29′S 73°10′W	19°38′N 54°19′W	19°40′N 48°52′W
Bact. Abund. (×10^6^ ml^−1^)	n/a^1^	0.20	0.46	0.58	0.40	0.38	0.81	0.82	0.24	0.24
Chl *a* content (µg L^−1^)	1.34	0.69	1.64	3.18	0.54	0.37	0.74	3.25	0.20	0.20
Temperature (°C)	−0.8	18.0	19.0	20.0	28.0	26.5	17.0	12.0	26.2	26.0

Samples were obtained from 5 m depth. ^1^n/a –not available.

Community DNA was extracted using an enzyme/phenol-chloroform protocol [11]. The Arctic sample was extracted from two size fractions (>3 µm and 0.2–3 µm; [12]). DNA was quantified using PicoGreen (Molecular Probes). The two size fractions of the Arctic sample were pooled at equal amounts. Community RNA was extracted using the RNeasy mini kit (Qiagen) and cDNA was synthesized using the TaqMan reverse transcription kit (Applied Biosystems). Four blank filters in TE buffer or RNAlater were extracted (two for DNA and two for RNA) exactly as described above as negative controls to ensure that amplifiable nucleic acids were not derived from the filters or the extraction reagents.

### PCR amplification and pyrosequencing

Equal volumes (1 µl) of the DNA extracts or cDNA reactions from samples or the negative extraction controls were added to PCR reactions to amplify the nitrogenase gene (*nifH*) according to a nested PCR protocol [13] using Pure Taq Ready-To-Go PCR Beads (GE Healthcare). The degenerate primers were purified by high-performance liquid chromatography and polyacrylamide gel electrophoresis. Non-transcribed RNA samples were included to ensure that cDNA amplicons were not products of incomplete digestion of DNA. To minimize the risk of contamination, pipettes and DNA-free filter tips were UV treated (>20 min) before use, mixing of PCR reagents was done in a UV-treated sterile flow bench, and DNA template was added in a PCR/UV workstation in a separate room. In addition to the four extraction controls, six negative PCR controls with UV-treated water instead of DNA template were included in the nested PCR batches and subjected to the exact same treatments as the samples; i.e. a total of 60 cycles. Products from triplicate PCR reactions were gel purified (Gel extraction kit, Qiagen), pooled, and concentrated (PCR purification kit, Qiagen) for subsequent tagging.

None of the ten negative controls resulted in a visible band when run on a gel. Nevertheless, a section of the gel encompassing the correct product size (∼359 bp) was identified using marker ladders and excised from each negative control lane. These gel fragments were processed like the samples using a gel extraction kit (Qiagen). DNA was not detectable in the extracts by spectrophotometry (NanoDrop 2000, Thermo Scientific) or by fluorometry (PicoGreen), so these negative controls were not further processed for pyrosequencing.

For 454 pyrosequencing of samples, adapters and sample-specific tags were added using custom primers (Table S1) in an additional PCR amplification of 10 cycles using the same PCR conditions [14]. The amplified fragments were quantified using a Qubit™ fluorometer (Invitrogen) and quantitative PCR (Mx-3000 thermocycler; Stratagene) as previously described [15], mixed in equal amounts, and sequenced in a two-region 454 run on a 70×75 GS PicoTiterPlate using a GS FLX pyrosequencing system according to manufacturer instructions (Roche). Pyrotag sequences have been deposited in the National Center for Biotechnology Information (NCBI) Sequence Read Archive under accession number SRP002157.

### Cloning and sequencing of negative controls

Although no DNA was detected in the amplification reactions from the negative controls, we wanted to determine whether *nifH* sequences might be present in trace amounts. Subsamples of the gel-purified extracts were therefore added to cloning reactions (TOPO TA cloning kit, Invitrogen). To ensure that the cloning reactions worked, a positive cloning control was included. Forty colonies resulted from these cloning reactions, all of which were sequenced (Macrogen, Korea). Only 16 of the 40 clones contained *nifH*-like inserts. Negative control sequences have been deposited in GenBank under accession numbers HM042878 to HM042893.

### Sequence quality control, removal of contaminant-like sequences, phylogenetic composition, and diversity

Primer sequences were trimmed and reads <200 bp long and sequences with undetermined nucleotides were removed [16]. Remaining sequences were clipped at 180 bp, translated into amino acid sequence, and sequences having in-frame stop codon(s) were removed. All subsequent analyses were done on amino acid sequences. Unique sequences were aligned with hmmalign (http://mobyle.pasteur.fr; [17]) using the profile Fer4_NifH_fs.hmm, and sequences with unaligned characters were removed. Thus, by targeting a protein coding gene, frame-shift errors caused by insertions or deletions of bases, commonly associated with the 454 pyrosequencing technique [16], can be distinguished and removed. Further, through clustering of sequences (Cd-hit; [18]), we prevent mismatch (substitution) errors that would otherwise be interpreted as true diversity [19]. However, as in taxonomic composition studies using pyrosequencing, artificial replicates [20] and PCR bias may have influenced the relative abundance of phylotypes within a sample.

One concern when targeting *nifH* is that *nifH*-like sequences related to *Alpha*- and *Betaproteobacteria* may occur in PCR reagents [21], [22]. Although none of our negative controls produced visible product, to reduce the risk of misinterpreting reagent contamination as true environmental templates, sequences of ≥96% amino acid identity (determined using NCBI Blast) to putative contaminant *nifH* sequences from this study and all known previous reports [21]–[25] were removed from the dataset. This precaution reduced the number of high quality reads from 117 440 to 79 090 particularly influencing the South Africa, Azores and Chile samples (97%, 82% and 27% of sequences removed, respectively). For the Sargasso Sea DNA2, Fiji and Australia samples 15–17% of the sequences were removed and for the remaining six samples ∼0% of the sequences were removed. Notably the similarity between samples (Table S2) was not greatly influenced by the removal of sequences. Unfortunately, in a PCR approach one cannot be certain that any particular sequence is not a contaminant; however, our removal of contaminant-like sequences was more conservative than applied in any previous similar study. It is likely that *nifH* sequences from some legitimate marine *Proteobacteria* have been removed.

To construct a phylogenetic tree in MEGA4 [26], *nifH* sequences were first clustered at 92% similarity (complete linkage clustering) and clusters with <10 sequences (together representing 0.9% of the dataset) were removed for clarity. Nearest relatives were retrieved using the NCBI search TBLASTN. A heatmap showing the relative number of sequences per sample for each cluster was added in iTol [27]. A pair-wise sample distance matrix, based on the same phylogenetic tree as above, was calculated with weighted UniFrac [28] and projected in two dimensional space using Principal Coordinates Analysis in *R* (www.r-project.org). To assess the compositional similarity between two samples, a 96% similarity clustering level was used and the Sørensen's index of similarity (C_s_) was calculated (Table S2). To normalize the relative abundance of sequences between samples, random re-sampling to identical sequencing depth was done using an in-house developed Perl script. A subsample of 2 883 sequences from the pool of re-sampled data sets (10×2 883 sequences) was also obtained, named “mixed” sample. Rarefaction curves were generated using Analytical Rarefaction 1.3 (http://www.uga.edu/strata/software/index.html; Figure S1). The S_Chao1_ richness estimator and the Shannon diversity index were calculated using the re-sampled datasets (Figure S2).

## Results and Discussion

**Figure 2:**
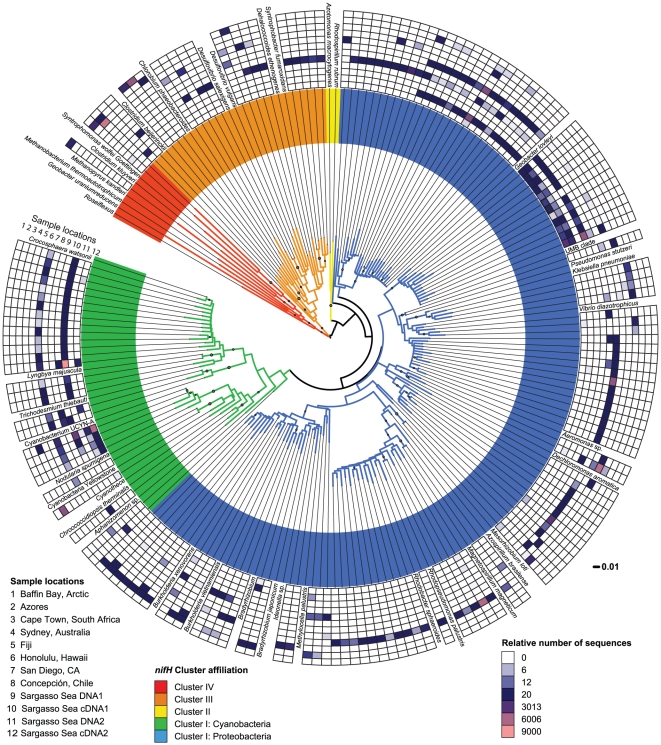
Phylogeny and relative composition of the sequenced *nifH* assemblages. Neighbor-joining phylogenetic tree of 92% similarity clustered *nifH* sequences (79 090) and nearest relatives from ten sampling locations. The relative number of sequences in each cluster is shown in a heatmap with the median value of the dataset indicated in dark blue and the highest value in pink. White indicates that there are no sequences from the cluster present in the sample. Bootstrap values (500 replicates) >50% are indicated with grey circles proportional to the size of the bootstrap value. Accession numbers of reference sequences, sequence codes for each cluster and the absolute number of sequences for each sample are shown in Figure S4.

PCR amplicons of a fragment of the *nifH* gene from twelve ocean samples were subjected to pyrosequencing and 79 090 reads representing 7 468 unique protein sequences, 60 amino acids in length, were analysed (Figure 1, Table 1). In comparison, GenBank, as of 3 January 2011, contained <1 500 *nifH* gene sequences from marine plankton samples collected more than 5 km from the shoreline (Table S3). These data thus represent a significant increase in the number of publicly available *nifH* sequences from offshore ocean waters. Since *nifH* and 16S rRNA gene-based phylogenies are largely congruent [4], bacterial identity can be inferred from *nifH* sequence information. The pyrosequencing chemistry used here only allowed for retrieval of partial *nifH* genes; however, phylogenetic trees based on short fragments from reference *nifH* genes (60 amino acids) compared to the length usually used for *nifH* phylogeny (108 amino acids) showed only few topological differences (Figure S3) and sequences from cultivated representatives clustered within previously designated *nifH* Clusters (Figures 2 and S4). This suggests that the analyzed fragments (60 amino acids) provide sufficient information for meaningful phylogenetic classification. The obtained sequences were distributed among the canonical *nifH* Clusters I, III and IV ([29]; Figures 1 and 2). Cluster I includes mainly *nifH* sequences from Cyanobacteria, *Alpha*-, *Beta*- and *Gammaproteobacteria*, Cluster III includes anaerobes, such as methanogens, *Clostridia*, and some *Deltaproteobacteria*, while Cluster IV includes diverse *nifH* homologs found in methanogens [4].

### Coverage, diversity, and similarity between samples

The number of high-quality sequences varied among samples from 477 to 15 290 (Figure 1). To allow for comparisons of diversity and richness among samples, a subsample of 2 883 random sequences was analyzed from each sample. The South Africa and Sargasso Sea DNA1 samples were excluded in these analyses because of their low number of sequences. The small differences between the Chao1 richness estimator and the observed number of clusters, as well as the near-plateau phase of the rarefaction curves (Figures S1 and S2), suggest that the *nifH* amplicon libraries covered local *in situ nifH* diversity well. In contrast, analysis of a “mixed” sample composed of 2 883 sequences randomly selected from the combined pool of *nifH* subsamples indicated that this sequencing depth was not sufficient to cover global *nifH* diversity (Figure S2).

The highest diversity was observed for the sample from waters off the Chilean coast, which was characterized by groups of clusters scattered across the phylogenetic tree, and lowest for the Sargasso Sea cDNA1 sample, which consisted of only one major group of clusters (Figures 2 and S4). It should be noted that the four Sargasso Sea samples were pre-filtered (10 µm pore size) to avoid dominance of filamentous cyanobacteria, probably affecting the *nifH* composition and diversity in these samples. Although diversity estimates in different studies are not directly comparable because of different sequencing depths and clustering levels, previously reported *nifH* clone libraries indicate high diversity in coastal and estuarine environments [24], [30], [31] while open ocean samples appear to have fewer *nifH* phylogenetic groups [4]. This may indicate a linkage between *nifH* diversity and nutrient status, which is consistent with our finding of highest *nifH* diversity and chl *a* in the Chile sample (Table 1).

In this study, three cut-off levels (100%, 96% and 92% amino acid similarity) were used for generating rarefaction curves, and diversity and richness estimates (Figures S1 and S2). A 92% cut-off was applied to allow for graphical presentation of the overall *nifH* phylogeny (Figures 1 and 2) while a 96% cut-off [32] was used to delimit *nifH* phylogenetic groups for analyses of similarity between the samples (Table S2, Figure S5). The number of clusters was greatly reduced when applying decreasing similarity cut-offs. This was particularly evident for the Arctic sample, which had 462 unique sequences, but only 13 clusters at 92% similarity (Figures S1 and S2). A large but variable microdiversity was observed for the different samples indicating unique phylogenetic structures (Figures S1 and S2). Intra-cluster microdiversity is thought to persist because of weak selection pressures [33]; however, it is unknown to what extent *nifH* genes are exposed to *in situ* selection pressure.

**Figure 3:**
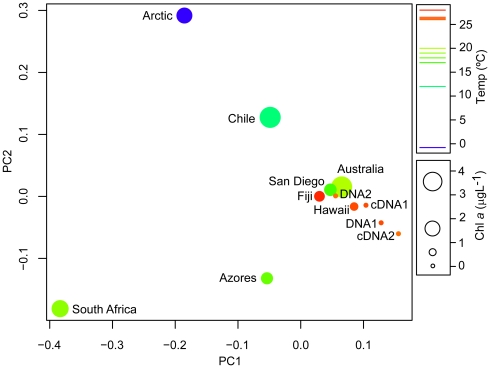
Principal Coordinate Analysis plot depicting *nifH* assemblage similarity between samples. The plot illustrates the compositional and phylogenetic similarity between samples based on weighted UniFrac. Temperature and chlorophyll (chl *a*) are indicated by color and size of the dots for each sample in the plot.

The *nifH* composition at the different sites showed great divergence, illustrated by low Sørensen similarity indices (Table S2) and few cluster overlaps between samples (Figures 2 and S5). Generally, samples characterized by low chl *a* concentration and high temperatures were most similar while the Arctic sample was an extreme outlier sharing no *nifH* phylotypes with the others (Figure 3, Table S2). The Sørensen similarity indices indicated that the Sargasso Sea samples, located only ∼5 km apart, were more similar (up to 72%, Table S2) compared to samples from other locations; however, the composition did vary considerably (Figures 2 and S4). For example, according to the UniFrac Principal Coordinate Analysis (PCoA), the Sargasso Sea DNA2 grouped with the Hawaii sample (Figure 3, Table S4). This was primarily due to the predominance of amplicons of a *nifH* Deltaproteobacterial group, which was almost absent in the other Sargasso Sea samples (Figure 2).

From what is known from cultivated organisms, presence of *nifH* mRNA is a reasonable indicator of active N_2_ fixation (e.g., [29], [34]). Therefore, to identify which of the present *nifH* phylotypes were active, the Sargasso Sea DNA and cDNA samples were compared. The most actively expressed *nifH* sequences were not the most prevalent in the DNA samples. For example, a rare cluster in the DNA1 sample (0.7% of sequences) was dominant in the cDNA1 sample (88% of sequences, Figure 2). This indicates that rare members of the diazotrophic community may at least occasionally be important for local N_2_ fixation. As anticipated, amplicons from the DNA samples were more diverse than amplicons from the respective cDNA samples (Figures S1 and S2). Moreover, unlike previous observations of few overlaps between DNA- and cDNA-based clone libraries (e.g., [9]), our deep sequencing revealed that a majority of the clusters detected in cDNA could be found in the corresponding DNA samples (Figures 2 and S4).

### Proteobacterial dominance in marine NifH amplicons

The DNA samples showed an overwhelming dominance of diverse *nifH* amplicons related to non-cyanobacteria, especially to *Proteobacteria* (Figure 1). This was the case even in tropical and subtropical waters, which are preferred by cyanobacterial diazotrophs [10]. The proportions of cyanobacterial amplicons in the Sargasso Sea samples were likely underestimated because of the removal of *Trichodesmium* by the applied 10 µm prefilter [8], [35], but none of the other samples were prefiltered. Due to bias inherent in any PCR, the relative representation of template genes among amplicons may be skewed particularly when applying a high number of thermal cycles as in the present approach [36], [37]. Although the composition of *nifH* amplicons may occasionally be similar to the relative distribution of *nifH* phylotypes as quantified by qPCR (e.g., [38]), large deviations for single phylotypes have also been observed [39]. Hence, one cannot take for granted that *nifH* amplicons always reflect true composition of *nifH* genes *in situ*. Nevertheless, we stress that the primers used in this study have few mismatches to complete *nifH* sequences available in GenBank (May 2006) and show no preferential amplification of *Proteobacteria* [40]. Moreover, in line with our sequencing results, we found that genes from non-cyanobacteria accounted for 53% of the *nifH* genes from offshore marine plankton available in GenBank (Table S3).

Despite frequent detection of *Proteobacteria*-like *nifH* genes in PCR amplicons as well as in real-time PCR based studies from open oceans [41]–[44], these putative diazotrophs have received limited attention, perhaps because open ocean waters that are well-oxygenated and poor in energy sources would appear to be unfavourable for expression of the nitrogenase genes [45]. Nevertheless, keeping in mind the nearly inexhaustible source of genomic innovation found in the bacterial world, exemplified by the recent discoveries of novel organisms and metabolic strategies playing prominent roles in the N cycle [7], [46], it can be expected that microorganisms other than cyanobacteria have developed strategies to overcome the chemical and physical challenges of fixing N_2_ in the pelagic oceans.

Indeed, this study and others [8], [9] show that at least some of the non-cyanobacterial *nifH* genes detected in seawater are expressed. Although no transcripts of non-cyanobacteria were found in the cDNA1 sample (Figure 1), 42% of the non-cyanobacterial clusters (Figure 2), representing 82% of the sequences, identified in the Sargasso Sea DNA2 sample were also detected in the cDNA2 sample (Figure S4). Even though a large portion of the identified Sargasso Sea non-cyanobacterial clusters were expressed these were almost exclusively associated with the UMB group (uncultured marine bacteria; [47]). Expression of some of the *Proteobacteria*-like *nifH* genes detected in surface oceans have yet to be observed; but it is likely that these are occasionally expressed since only genes providing selective advantages are expected to be fixed in populations [48]. Accordingly, we were unable to amplify *nifH* genes from multiple stations in Antarctic waters; locations, which are consistently N-replete and where a capacity for N_2_ fixation is unlikely to confer a selective advantage. The absence of some *nifH* phylotypes from cDNA libraries may, therefore, simply reflect the fact that gene expression is transient.

The proteobacterial *nifH* amplicons consisted of diverse classes, and pronounced differences were observed between locations (Figure 2). Sequences clustering with the Betaproteobacterium *Dechloromonas aromatica* (98% similarity, Figure 2) accounted for 68% of the sequences in the Fiji sample and also occurred in the Australia, Hawaii, Chile, and Sargasso Sea DNA2 samples. Interestingly, the sequences were >96% similar to a *nifH* transcript from the Pacific Ocean [32] indicating that organisms carrying these *nifH* genes are widespread in marine waters and potentially active N_2_-fixers. The Gammaproteobacterial UMB group was detected in the Sargasso Sea DNA1, DNA2 and cDNA2, Chile and Hawaii samples (98% similarity, Figure 2) suggesting that these widespread bacteria are active players in local N_2_ fixation. This transcriptionally active group has previously been detected in the Pacific and Atlantic Oceans, and the Arabian and South China Seas [41], [43], [44], [47], and appears as a key group among non-cyanobacterial diazotrophs which has also been targeted by qPCR [41]. The San Diego sample consisted of groups of clusters within *Gammaproteobacteria*, which were mostly unique to this sample. Interestingly, a *Pseudomonas*-like cluster was 98% similar to *nifH* genes from the Red Sea [42] and *nifH* genes and transcripts from the Pacific Ocean [41], [49] indicating that this cluster may also be globally distributed and active.

The most prevalent amplicons in the Hawaii and Sargasso Sea DNA2 samples (45% and 44% of sequences, respectively) was 93–96% similar to *Geobacter* species (*Deltaproteobacteria*; Figure 2). *NifH* sequences and transcripts within this genus have also been reported from estuarine environments [30], [50]. The oxygen tolerance may give these bacteria a competitive edge at oxic-anoxic boundaries [51], facilitating growth and N_2_ fixation in low oxygen microzones (e.g. within particles) in the marine water column. Amplicons from the Australia sample were dominated by 17 clusters (75% of sequences) related to facultative anaerobic *Alphaproteobacteria* including the facultative phototrophic diazotrophs *Rhodopseudomonas palustris* and *Rhodobacter spaeroides* (Figure 2; [52]). Similarly, *nifH* genes related to these organisms have been detected in an Australian reef lagoon [53] and the South China Sea [44]. Interestingly, some heterotrophic marine bacteria may use light-driven proton pumps to complement ATP production providing critical amounts of energy for growth in oligotrophic environments [54]. Thus, an intriguing possibility is that some marine non-cyanobacterial diazotrophs could acquire energy through phototrophy to help meet the substantial energy demands associated with N_2_ fixation.

### NifH composition and expression in cyanobacteria

Cyanobacterial *nifH* sequences were found in all samples (except for the Arctic) and were mainly made up of groups thought to be important for N_2_ fixation in tropical and subtropical waters. Cyanobacteria of the genus *Trichodesmium* were detected in the Hawaii, Chile and Sargasso Sea DNA2 samples. Amplicons of unicellular cyanobacteria, related to group A (uncultured, also known as UCYN-A) or group B (93–100% *nifH* nucleotide similarity to *Crocosphaera watsonii*; [7]) were most prevalent in the Sargasso Sea cDNA samples (stations 2 and 1, respectively) and were also detected in the Hawaii sample (Figure 2). In contrast to the DNA samples, where non-cyanobacterial *nifH* amplicons were most prevalent, the cDNA samples (sampled at sunset) were dominated by *nifH* genes affiliated with cyanobacteria (Figure 1) suggesting a dominant role for cyanobacteria in local N_2_-fixation at this time and location.

A cluster of cyanobacterial amplicons was related to *C. watsonii* (89% of Sargasso Sea cDNA1 and 16% of Hawaii sequences) and was 98% similar to a sequence from a Sargasso Sea enrichment culture [49]. Interestingly, 13 clusters (<92% similarity clustering in Cd-hit) of comparable relative abundance (∼0.4% of sample for each cluster, Figures 2 and S2) also clustered with *C. watsonii*. This suggests a divergence of *C. watsonii* related *nifH* genes, which is in contrast to the high level of conservation previously observed for *C. watsonii* [55]. Such divergence was, however, not observed for UCYN-A, which only included four clusters on a well supported branch (Figure 2), with one cluster being particularly dominant (19% of Hawaii, 37% of Sargasso Sea DNA1, 16% of DNA2 and 63% of cDNA2 sequences). Interestingly, UCYN-A phylotypes lack genes coding for PS-II, but possess those for PS-I, suggesting photoheterotrophic energy acquisition [46]. The prevalence of these sequences in the samples from Sargasso Sea and Hawaii suggests that this growth strategy may be particularly advantageous in oligotrophic tropical waters.

### Prevalence of nifH Clusters III and IV

Diverse Cluster III and IV phylotypes were detected in eight samples (Figure 2). Sequences affiliated with *nifH* Cluster III, consisting of diverse anaerobes, have been detected [56] but appear uncommon in the surface ocean [38], [43]. Similarly, few sequences belonging to Cluster IV, consisting of divergent and non-functional *nifH*-like homologues, have been reported [4]. We speculate that our finding of Cluster III and IV sequences widely distributed in the surface ocean is a consequence of the increased sequencing depth offered by pyrosequencing relative to conventional clone libraries. Interestingly, the Arctic sample exclusively contained *nifH* sequences that were distantly related to known phylotypes (63–81% similarity) within Clusters III and IV. The presence of unique putative diazotrophs in the Arctic (Figure 2) is consistent with the finding of 16S rRNA gene phylotypes endemic to polar regions [57], but we do not know anything yet about the ecology or physiology of these distinct *nifH* phylotypes that would suggest why they prevail in the Arctic Ocean.

In total, 20 clusters (Figure 2), representing six samples (Arctic, Azores, Australia, Hawaii, Chile and Sargasso DNA2), affiliated with *nifH* Cluster III. Most of these were distantly related to anaerobes such as *Chlorobium* and *Desulfovibrio*. *NifH* amplicons related to Cluster III were particularly prevalent in the Arctic and Chile samples, accounting for 86% and 42% of the sequences, respectively (Figure 1). The presence of *nifH* sequences related to anaerobic microorganisms suggests that anoxic microsites exist in the oxygenated water column. It has been suggested that these bacteria thrive on particles or in association with zooplankton [9], [41], [45], [58]. However, their presence in prefiltered surface samples (Sargasso DNA2; [24], [41]) as well indicates that they may be facultative anaerobes thriving even in the fully oxygenated free-living phase. The predominance of Cluster III amplicons in the Arctic sample and high prevalence in the Chile sample may point to a preference for low temperature. Three clusters affiliating with *nifH* Cluster IV were represented in four samples (Arctic, Azores, South Africa and Sargasso Sea cDNA1; Figure 2). The primers used here have many mismatches to known Cluster IV phylotypes [40]; hence, we may have underestimated the prevalence of Cluster IV.

### Conclusions

This first in-depth sequencing analysis of *nifH* genes in marine surface waters generated a wealth of new data and novel insights into the diversity and composition of diazotrophic plankton. The *nifH* amplicons showed great spatial divergence in composition, but were frequently dominated by genes from taxonomically diverse organisms other than cyanobacteria, particularly of clusters related to Alpha-, Beta-, Gamma-, and Delta-Proteobacteria, and at least some of these were expressed at the time of sampling. Future quantification is needed to address temporal and spatial dynamics of key *nifH* phylotypes in marine waters; however, our reporting of the preponderance of these types of *nifH* sequences among amplicons from plankton samples throughout the world ocean is a stark reminder that we still know very little about these putative non-cyanobacterial diazotrophs in the sea. Our data suggest that an accounting of their ecology, physiology, and N_2_ fixation rates is not only worthwhile, but will contribute to a better understanding of the complexity of the marine N cycle.

## Supporting Information

Figure S1 **Rarefaction curves of** ***nifH*** **sequence libraries.** Curves of sub-sampled datasets (2 883 random sequences per sample) clustered based on (A) 100%, (B) 96% and (C) 92% similarity cut-offs.
(TIF)

Figure S2 **Sample richness and diversity.** Number of unique sequences or number of clusters, S_Chao1_ [59] richness estimator and Shannon [60] diversity indices at (A) 100%, (B) 96% and (C) 92% amino acid similarity levels for sub-sampled samples (2 883 sequences each) and a mixed sample composed of 2 883 random sequences from the sub-sampled dataset (10×2 883 sequences). The Sargasso Sea DNA1 and South Africa samples were excluded from the analyses due to the small sample size. Note different scales on Y-axes.
(TIF)

Figure S3 **Phylogeny based on “full” or partial** ***nifH*** **gene segments.** Comparison of *nifH* neighbor-joining phylogenetic trees constructed with the full gene segment amplified by nifH1 and nifH2 primers (108 amino acids; to the left) and the partial gene segment obtained using 454 pyrosequencing (60 amino acids; to the right) starting directly downstream from the nifH1 primer. The trees are constructed based on reference sequences with the corresponding accession numbers or GenInfo Identifier (GI) shown in brackets and within vertical bars, respectively. Bootstrap values (500 replicates) >50% are indicated by numbers.
(TIF)

Figure S4 **Phylogeny and composition of the sequenced** ***nifH*** **assemblages.** Neighbor-joining phylogenetic tree of 92% clustered *nifH* amino acid sequences (79 090 sequences) from ten sampling locations world-wide and nearest relatives in GenBank. The figure provides information about the number of sequences per cluster for each sample (table grid), sequence codes of *nifH* clusters detected in this study, and accession numbers or GenInfo Identifier (GI) of reference sequences (in brackets and vertical bars, respectively). A blank square indicates that the cluster is not present in the sample. Bootstrap values (500 replicates) >50% are indicated with grey circles proportional to the size of the bootstrap value. Clusters with <10 sequences (representing 0.9% of the dataset) have been removed for clarity purpose.
(PDF)

Figure S5 **Degree of cluster overlap between the 12 samples based on 96% amino acid similarity.**
(TIF)

Table S1 **Custom primers.** Custom primers including nifH1 and nifH2 (bold; [61]), adaptor and sample-specific tags (underlined) used for pyrosequencing in this study.
(DOC)

Table S2 **Sørensen similarity index.** Sørensen similarity index in percent of (A) the total *nifH* dataset (117 440 sequences) and (B) the *nifH* dataset after removal of contaminant-like sequences (79 090 sequences). ^1^Number of *nifH* clusters at 96% similarity. The index (Cs) was calculated as 2j/a+b * 100, where j is the number of common clusters between the samples and a and b are the number of clusters in each sample [62].
(DOC)

Table S3 **Composition of** ***nifH*** **sequences in GenBank obtained from offshore marine samples.** Numbers and relative proportions of *nifH* sequences from marine plankton DNA or RNA samples that are most similar to cyanobacteria (Cyano) or to sequences from other types of bacteria or archaea (Non-Cyano). The numbers in this table were derived from analysis of an Arb [63] database of aligned *nifH* sequences (http://www.es.ucsc.edu/~wwwzehr/research/database/) which was updated January 3, 2011. The database was manually curated to identify sequences derived from marine plankton samples collected from seawater at least 5 km from shore. Data are presented separately for sequences retrieved from diazotrophs associated with individually picked eukaryotic plankton (diatoms, dinoflagellates, and copepods). The affiliations of these marine offshore plankton samples were determined by their position relative to known microorganisms in a neighbor-joining tree constructed in Arb using a filter to include only amino acid residues 46–135 (*Azotobacter vinelandii* numbering). Sources include the Atlantic, Pacific, and Indian Ocean, the Mediterranean Sea, and the Gulf of Mexico. Sequences from estuaries, or offshore sites specifically identified as being influenced by a river plume were excluded, as were sequences from studies that specifically targeted a subset of N_2_-fixing microorganisms, or those which did not span the targeted region of the protein. We attempted to exclude *nifH* sequences from possible contaminants deriving from PCR reagents by removing all sequences that clustered (>90% similar) with sequences identified as deriving from PCR negative controls.
(DOC)

Table S4 **Distance matrix showing pair-wise similarities of** ***nifH*** **assemblages.** Unifrac distance matrix based on 92% similarity clustering of all sequences (79 090 sequences) and removal of clusters with <10 sequences.
(DOC)
