# Supplementary figures and images for: Nitrogenase Gene Amplicons from Global Marine Surface Waters Are Dominated by Genes of Non-Cyanobacteria
Source: PLoS One. 2011 Apr 29;6(4):e19223. doi: 10.1371/journal.pone.0019223 (PMC3084785; doi:10.1371/journal.pone.0019223)

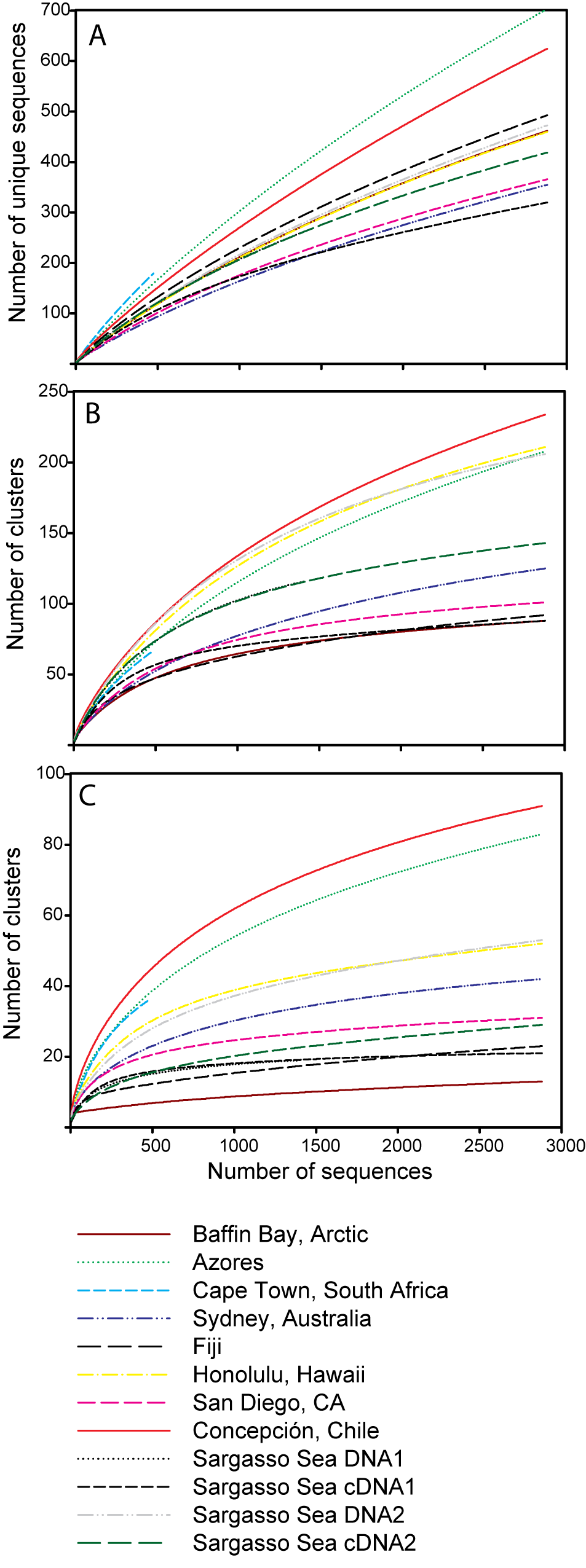

Supplement: Figure S1 — Rarefaction curves of nifH sequence libraries. Curves of sub-sampled datasets (2 883 random sequences per sample) clustered based on (A) 100%, (B) 96% and (C) 92% similarity cut-offs. (TIF) [file pone.0019223.s001.tif]

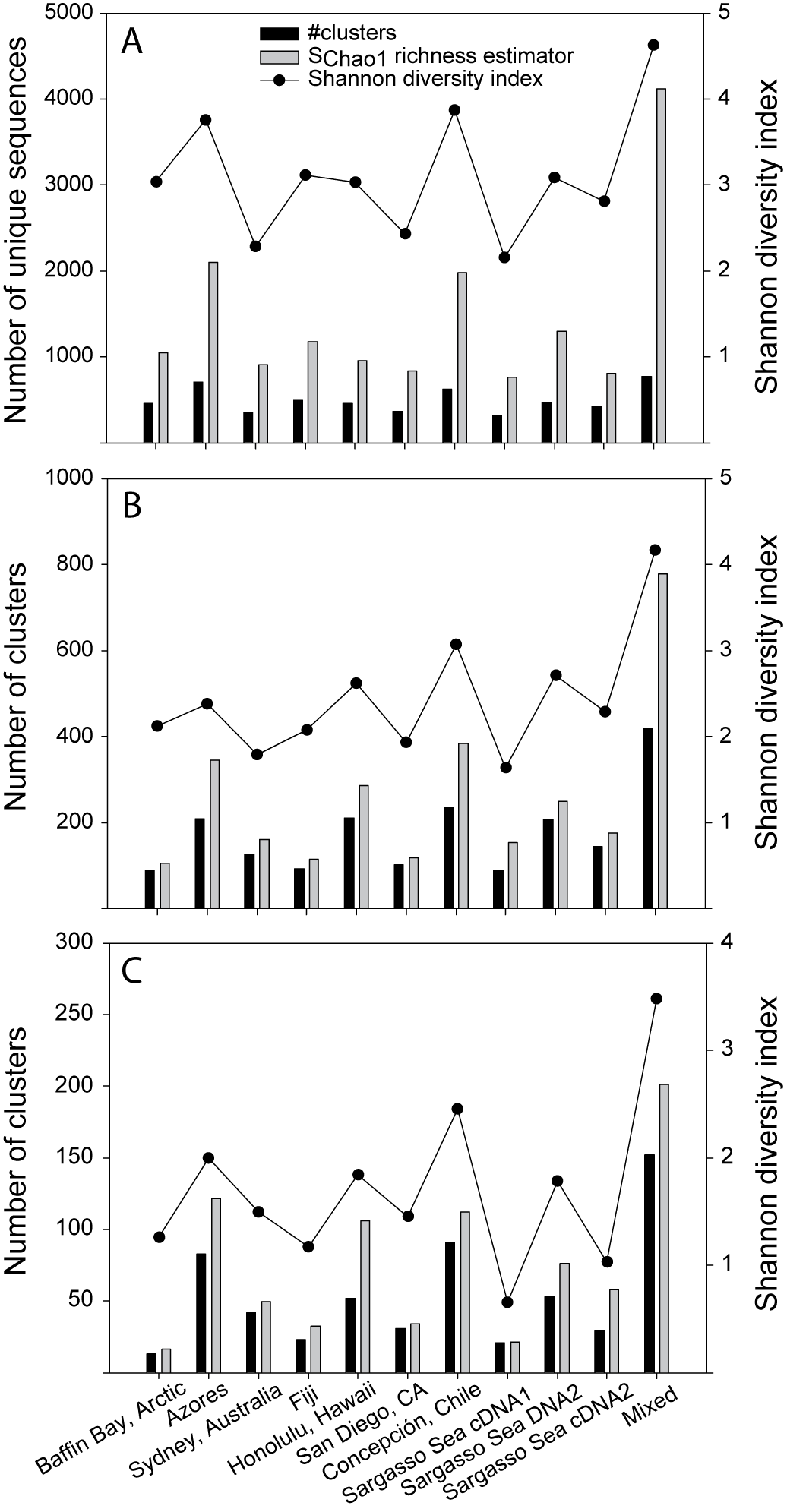

Supplement: Figure S2 — Sample richness and diversity. Number of unique sequences or number of clusters, SChao1 [59] richness estimator and Shannon [60] diversity indices at (A) 100%, (B) 96% and (C) 92% amino acid similarity levels for sub-sampled samples (2 883 sequences each) and a mixed sample composed of 2 883 random sequences from the sub-sampled dataset (10×2 883 sequences). The Sargasso Sea DNA1 and South Africa samples were excluded from the analyses due to the small sample size. Note different scales on Y-axes. (TIF) [file pone.0019223.s002.tif]

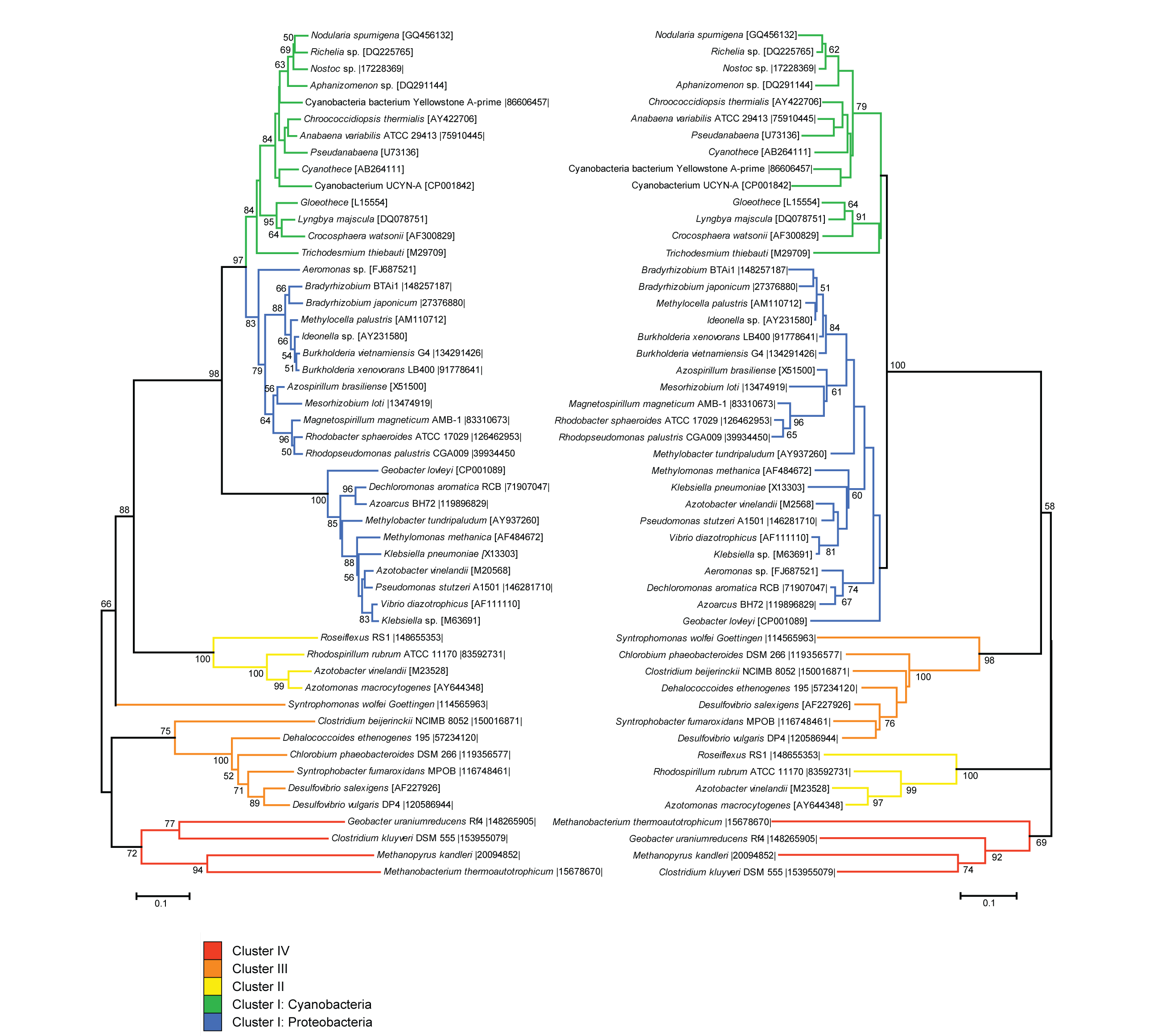

Supplement: Figure S3 — Phylogeny based on “full” or partial nifH gene segments. Comparison of nifH neighbor-joining phylogenetic trees constructed with the full gene segment amplified by nifH1 and nifH2 primers (108 amino acids; to the left) and the partial gene segment obtained using 454 pyrosequencing (60 amino acids; to the right) starting directly downstream from the nifH1 primer. The trees are constructed based on reference sequences with the corresponding accession numbers or GenInfo Identifier (GI) shown in brackets and within vertical bars, respectively. Bootstrap values (500 replicates) >50% are indicated by numbers. (TIF) [file pone.0019223.s003.tif]

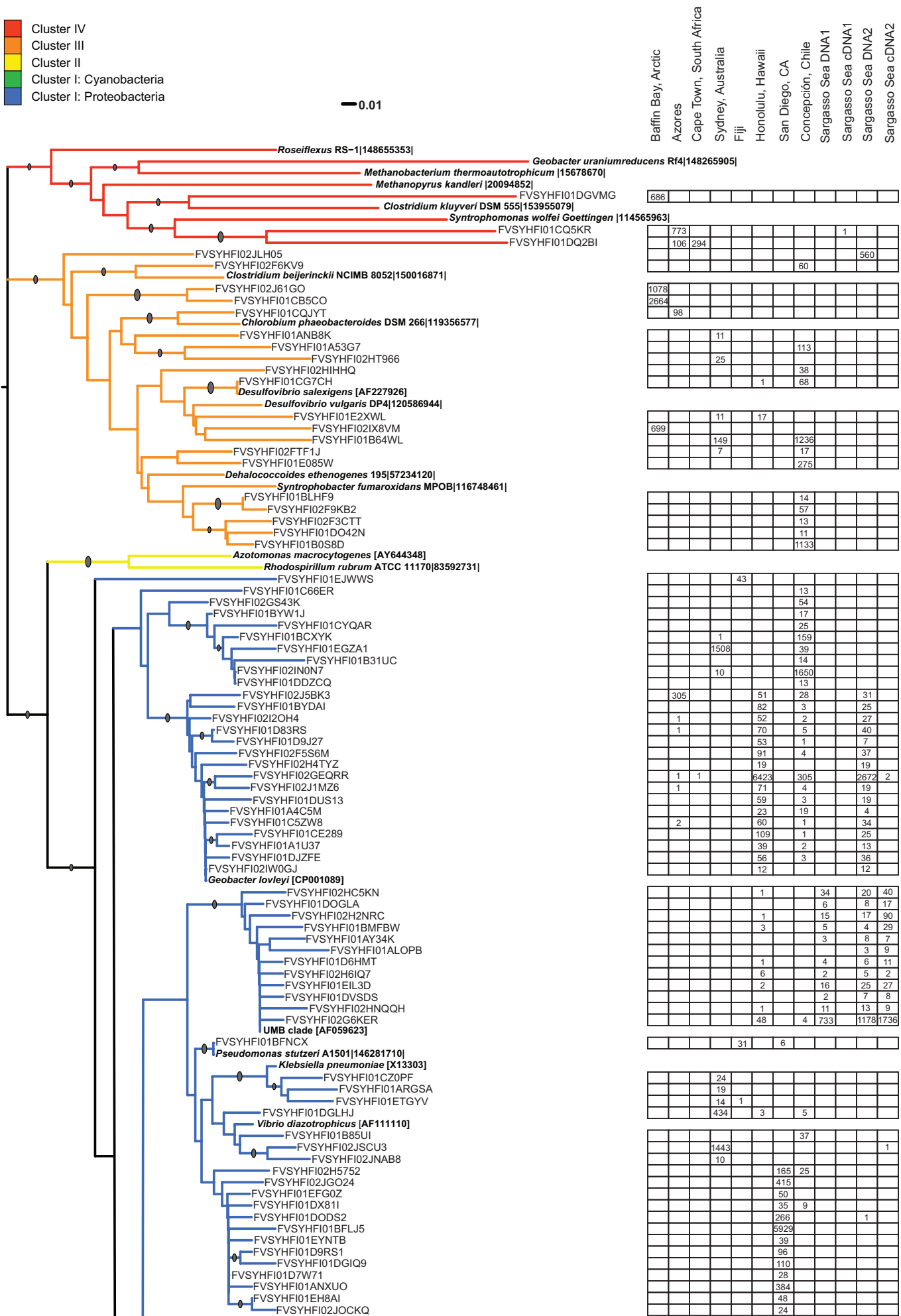

continued from previous page

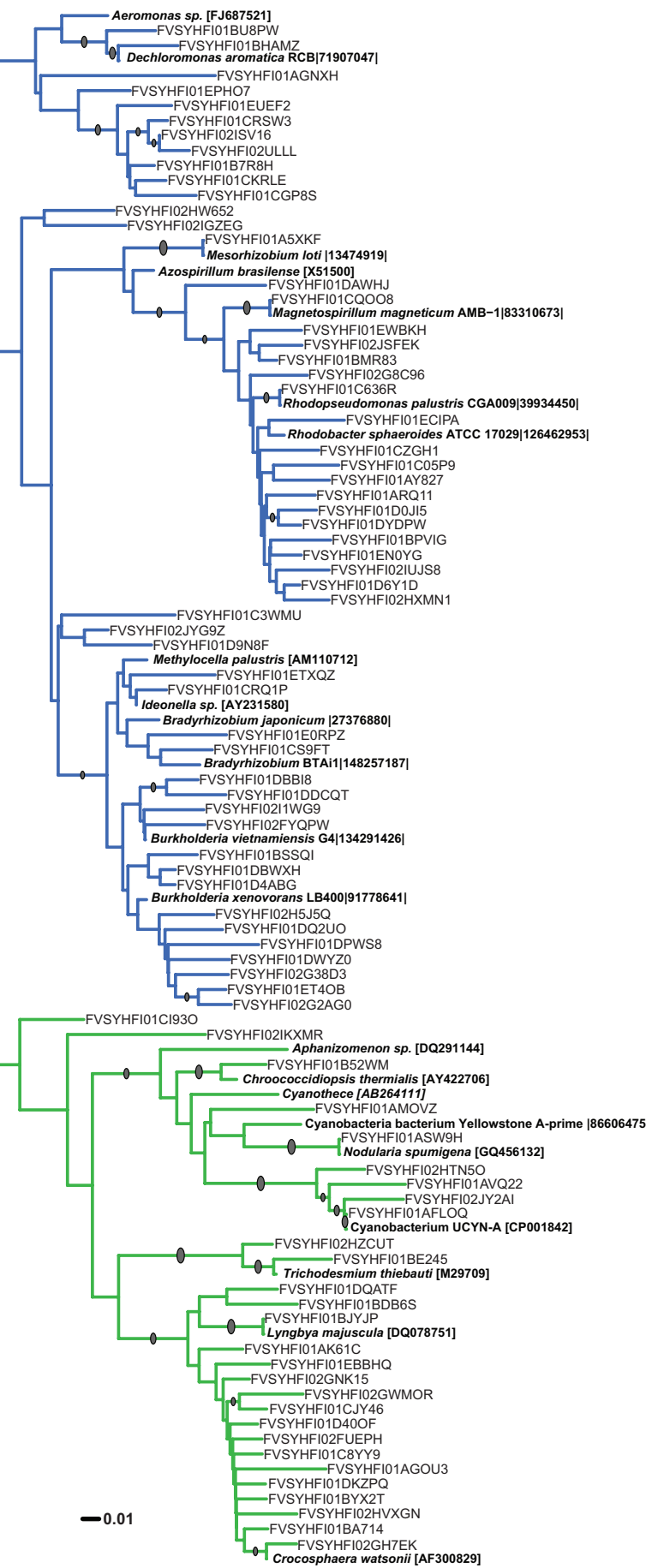[illegible]

Supplement: Figure S4 — Phylogeny and composition of the sequenced nifH assemblages. Neighbor-joining phylogenetic tree of 92% clustered nifH amino acid sequences (79 090 sequences) from ten sampling locations world-wide and nearest relatives in GenBank. The figure provides information about the number of sequences per cluster for each sample (table grid), sequence codes of nifH clusters detected in this study, and accession numbers or GenInfo Identifier (GI) of reference sequences (in brackets and vertical bars, respectively). A blank square indicates that the cluster is not present in the sample. Bootstrap values (500 replicates) >50% are indicated with grey circles proportional to the size of the bootstrap value. Clusters with <10 sequences (representing 0.9% of the dataset) have been removed for clarity purpose. (PDF) [file pone.0019223.s004.pdf]

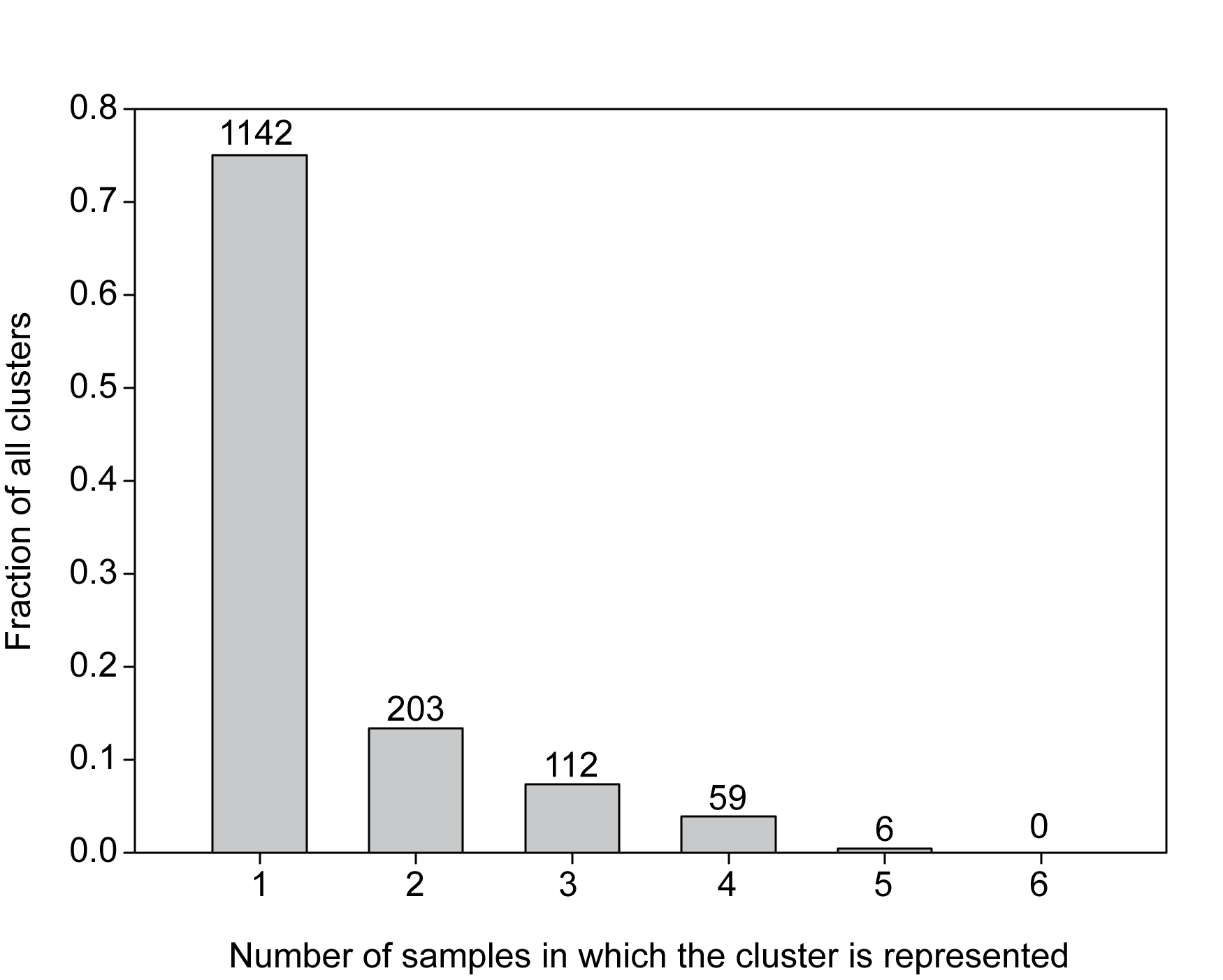

Supplement: Figure S5 — Degree of cluster overlap between the 12 samples based on 96% amino acid similarity. (TIF) [file pone.0019223.s005.tif]
