# Supplementary material for: Nitrogenase Gene Amplicons from Global Marine Surface Waters Are Dominated by Genes of Non-Cyanobacteria
Source: PLoS One. 2011 Apr 29;6(4):e19223. doi: 10.1371/journal.pone.0019223 (PMC3084785; doi:10.1371/journal.pone.0019223)
Supplement: Table S1 — Custom primers. Custom primers including nifH1 and nifH2 (bold; [61]), adaptor and sample-specific tags (underlined) used for pyrosequencing in this study. (DOC) [file pone.0019223.s006.doc]

| Name | Primer sequence |
| --- | --- |
| >LinA_nifH1F_1 | GCCTCCCTCGCGCCATCAGACGAGTGCGT**TGYGAYCCNAARGCNGA** |
| >LinA_nifH1F_2 | GCCTCCCTCGCGCCATCAGACGCTCGACA**TGYGAYCCNAARGCNGA** |
| >LinA_nifH1F_3 | GCCTCCCTCGCGCCATCAGAGACGCACTC**TGYGAYCCNAARGCNGA** |
| >LinA_nifH1F_4 | GCCTCCCTCGCGCCATCAGAGCACTGTAG**TGYGAYCCNAARGCNGA** |
| >LinA_nifH1F_5 | GCCTCCCTCGCGCCATCAGATCAGACACG**TGYGAYCCNAARGCNGA** |
| >LinA_nifH1F_6 | GCCTCCCTCGCGCCATCAGATATCGCGAG**TGYGAYCCNAARGCNGA** |
| >LinA_nifH1F_7 | GCCTCCCTCGCGCCATCAGCGTGTCTCTA**TGYGAYCCNAARGCNGA** |
| >LinA_nifH1F_8 | GCCTCCCTCGCGCCATCAGCTCGCGTGTC**TGYGAYCCNAARGCNGA** |
| >LinA_nifH1F_9 | GCCTCCCTCGCGCCATCAGTAGTATCAGC**TGYGAYCCNAARGCNGA** |
| >LinA_nifH1F_10 | GCCTCCCTCGCGCCATCAGTCTCTATGCG**TGYGAYCCNAARGCNGA** |
| >LinA_nifH1F_11 | GCCTCCCTCGCGCCATCAGTGATACGTCT**TGYGAYCCNAARGCNGA** |
| >LinA_nifH1F_12 | GCCTCCCTCGCGCCATCAGCATAGTAGTG**TGYGAYCCNAARGCNGA** |
| >LinB_nifH2R | GCCTTGCCAGCCCGCTCAG**ADNGCCATCATYTCNCC** |
