# Supplementary material for: Nitrogenase Gene Amplicons from Global Marine Surface Waters Are Dominated by Genes of Non-Cyanobacteria
Source: PLoS One. 2011 Apr 29;6(4):e19223. doi: 10.1371/journal.pone.0019223 (PMC3084785; doi:10.1371/journal.pone.0019223)
Supplement: Table S2 — Sørensen similarity index. Sørensen similarity index in percent of (A) the total nifH dataset (117 440 sequences) and (B) the nifH dataset after removal of contaminant-like sequences (79 090 sequences). 1Number of nifH clusters at 96% similarity. The index (Cs) was calculated as 2j/a+b * 100, where j is the number of common clusters between the samples and a and b are the number of clusters in each sample [62]. (DOC) [file pone.0019223.s007.doc]

| A  Sample location (*n*^1^) | Baffin Bay, Arctic | Azores | Cape Town, South Africa | Sydney, Australia | Fiji | Honolulu, Hawaii | San Diego, CA | Concepción, Chile | Sargasso Sea DNA1 | Sargasso Sea cDNA1 | Sargasso Sea DNA2 | Sargasso Sea cDNA2 |
| --- | --- | --- | --- | --- | --- | --- | --- | --- | --- | --- | --- | --- |
| Baffin Bay, Arctic (101) | 100 |  |  |  |  |  |  |  |  |  |  |  |
| Azores (332) | 0 | 100 |  |  |  |  |  |  |  |  |  |  |
| Cape Town, South Africa (159) | 0 | 26 | 100 |  |  |  |  |  |  |  |  |  |
| Sydney, Australia (242) | 0 | 0 | 0 | 100 |  |  |  |  |  |  |  |  |
| Fiji (111) | 0 | 3 | 0 | 10 | 100 |  |  |  |  |  |  |  |
| Honolulu, Hawaii (335) | 0 | 5 | 1 | 1 | 0 | 100 |  |  |  |  |  |  |
| San Diego, CA (131) | 0 | 0 | 0 | 1 | 0 | 0 | 100 |  |  |  |  |  |
| Concepción, Chile (360) | 0 | 15 | 6 | 11 | 6 | 17 | 1 | 100 |  |  |  |  |
| Sargasso Sea DNA1 (118) | 0 | 0 | 1 | 0 | 0 | 33 | 0 | 3 | 100 |  |  |  |
| Sargasso Sea cDNA1 (96) | 0 | 1 | 1 | 1 | 0 | 35 | 1 | 0 | 5 | 100 |  |  |
| Sargasso Sea DNA2 (285) | 0 | 15 | 0 | 1 | 2 | 48 | 0 | 16 | 45 | 1 | 100 |  |
| Sargasso Sea cDNA2 (173) | 0 | 2 | 2 | 1 | 2 | 37 | 2 | 2 | 71 | 4 | 50 | 100 |

| B  Sample location (*n*^1^) | Baffin Bay, Arctic | Azores | Cape Town, South Africa | Sydney, Australia | Fiji | Honolulu, Hawaii | San Diego, CA | Concepción, Chile | Sargasso Sea DNA1 | Sargasso Sea cDNA1 | Sargasso Sea DNA2 | Sargasso Sea cDNA2 |
| --- | --- | --- | --- | --- | --- | --- | --- | --- | --- | --- | --- | --- |
| Baffin Bay, Arctic (101) | 100 |  |  |  |  |  |  |  |  |  |  |  |
| Azores (332) | 0 | 100 |  |  |  |  |  |  |  |  |  |  |
| Cape Town, South Africa (159) | 0 | 3 | 100 |  |  |  |  |  |  |  |  |  |
| Sydney, Australia (242) | 0 | 0 | 1 | 100 |  |  |  |  |  |  |  |  |
| Fiji (111) | 0 | 0 | 0 | 1 | 100 |  |  |  |  |  |  |  |
| Honolulu, Hawaii (335) | 0 | 6 | 1 | 1 | 0 | 100 |  |  |  |  |  |  |
| San Diego, CA (131) | 0 | 0 | 0 | 1 | 0 | 0 | 100 |  |  |  |  |  |
| Concepción, Chile (360) | 0 | 5 | 1 | 12 | 3 | 18 | 1 | 100 |  |  |  |  |
| Sargasso Sea DNA1 (118) | 0 | 0 | 1 | 0 | 0 | 32 | 0 | 3 | 100 |  |  |  |
| Sargasso Sea cDNA1 (96) | 0 | 1 | 0 | 1 | 0 | 35 | 1 | 0 | 5 | 100 |  |  |
| Sargasso Sea DNA2 (285) | 0 | 11 | 2 | 1 | 1 | 50 | 1 | 14 | 48 | 1 | 100 |  |
| Sargasso Sea cDNA2 (173) | 0 | 1 | 2 | 1 | 1 | 37 | 2 | 1 | 72 | 4 | 53 | 100 |
