# Supplementary material for: Nitrogenase Gene Amplicons from Global Marine Surface Waters Are Dominated by Genes of Non-Cyanobacteria
Source: PLoS One. 2011 Apr 29;6(4):e19223. doi: 10.1371/journal.pone.0019223 (PMC3084785; doi:10.1371/journal.pone.0019223)
Supplement: Table S3 — Composition of nifH sequences in GenBank obtained from offshore marine samples. Numbers and relative proportions of nifH sequences from marine plankton DNA or RNA samples that are most similar to cyanobacteria (Cyano) or to sequences from other types of bacteria or archaea (Non-Cyano). The numbers in this table were derived from analysis of an Arb [63] database of aligned nifH sequences (http://www.es.ucsc.edu/~wwwzehr/research/database/) which was updated January 3, 2011. The database was manually curated to identify sequences derived from marine plankton samples collected from seawater at least 5 km from shore. Data are presented separately for sequences retrieved from diazotrophs associated with individually picked eukaryotic plankton (diatoms, dinoflagellates, and copepods). The affiliations of these marine offshore plankton samples were determined by their position relative to known microorganisms in a neighbor-joining tree constructed in Arb using a filter to include only amino acid residues 46–135 (Azotobacter vinelandii numbering). Sources include the Atlantic, Pacific, and Indian Ocean, the Mediterranean Sea, and the Gulf of Mexico. Sequences from estuaries, or offshore sites specifically identified as being influenced by a river plume were excluded, as were sequences from studies that specifically targeted a subset of N2-fixing microorganisms, or those which did not span the targeted region of the protein. We attempted to exclude nifH sequences from possible contaminants deriving from PCR reagents by removing all sequences that clustered (>90% similar) with sequences identified as deriving from PCR negative controls. (DOC) [file pone.0019223.s008.doc]

| **Source** | **Target** | **Cyano** | **Non-Cyano** | **Total** | **% Non-Cyano** |
| --- | --- | --- | --- | --- | --- |
| Oceans and Seas (Offshore) | DNA | 596 | 546 | 1142 | 48% |
|  | RNA | 49 | 103 | 152 | 68% |
| Associated with Eukaryotic Plankton | DNA | 15 | 83 | 98 | 85% |
|  | **Total** | **660** | **732** | **1392** | **53%** |
