# Supplementary material for: Nitrogenase Gene Amplicons from Global Marine Surface Waters Are Dominated by Genes of Non-Cyanobacteria
Source: PLoS One. 2011 Apr 29;6(4):e19223. doi: 10.1371/journal.pone.0019223 (PMC3084785; doi:10.1371/journal.pone.0019223)
Supplement: Table S4 — Distance matrix showing pair-wise similarities of nifH assemblages. Unifrac distance matrix based on 92% similarity clustering of all sequences (79 090 sequences) and removal of clusters with <10 sequences. (DOC) [file pone.0019223.s009.doc]

| . | Baffin, Bay Arctic | Azores | Cape Town, South Africa | Sydney, Australia | Fiji | Honolulu, Hawaii | San Diego, CA | Concepción, Chile | Sargasso Sea DNA1 | Sargasso Sea cDNA1 | Sargasso Sea DNA2 | Sargasso Sea cDNA2 |
| --- | --- | --- | --- | --- | --- | --- | --- | --- | --- | --- | --- | --- |
| Baffin Bay, Arctic | 0 |  |  |  |  |  |  |  |  |  |  |  |
| Azores | 0.48 | 0 |  |  |  |  |  |  |  |  |  |  |
| Cape Town, South Africa | 0.54 | 0.45 | 0 |  |  |  |  |  |  |  |  |  |
| Sydney, Australia | 0.48 | 0.42 | 0.54 | 0 |  |  |  |  |  |  |  |  |
| Fiji | 0.43 | 0.37 | 0.49 | 0.25 | 0 |  |  |  |  |  |  |  |
| Honolulu, Hawaii | 0.45 | 0.30 | 0.52 | 0.31 | 0.27 | 0 |  |  |  |  |  |  |
| San Diego, CA | 0.42 | 0.37 | 0.49 | 0.26 | 0.20 | 0.25 | 0 |  |  |  |  |  |
| Concepción, Chile | 0.35 | 0.37 | 0.50 | 0.36 | 0.33 | 0.28 | 0.32 | 0 |  |  |  |  |
| Sargasso Sea DNA1 | 0.47 | 0.32 | 0.55 | 0.32 | 0.27 | 0.18 | 0.21 | 0.34 | 0 |  |  |  |
| Sargasso Sea cDNA1 | 0.47 | 0.35 | 0.55 | 0.33 | 0.27 | 0.22 | 0.26 | 0.34 | 0.26 | 0 |  |  |
| Sargasso Sea DNA2 | 0.42 | 0.32 | 0.50 | 0.30 | 0.26 | 0.10 | 0.24 | 0.27 | 0.19 | 0.29 | 0 |  |
| Sargasso Sea cDNA2 | 0.50 | 0.32 | 0.57 | 0.35 | 0.30 | 0.22 | 0.26 | 0.37 | 0.09 | 0.26 | 0.21 | 0 |
